# Exome sequencing identifies a de novo mutation of *CTNNB1* gene in a patient mainly presented with retinal detachment, lens and vitreous opacities, microcephaly, and developmental delay: Case report and literature review

**DOI:** 10.1097/MD.0000000000006914

**Published:** 2017-05-19

**Authors:** Niu Li, Yufei Xu, Guoqiang Li, Tingting Yu, Ru-en Yao, Xiumin Wang, Jian Wang

**Affiliations:** a Molecular Diagnostic Laboratory; b Department of Endocrinology and Metabolism, Shanghai Children's Medical Center, Shanghai Jiaotong University School of Medicine, Shanghai, China.

**Keywords:** *CTNNB1* gene, de novo mutation, developmental delay, microcephaly, ophthalmic phenotype

## Abstract

**Rationale:**

The *CTNNB1* (β-catenin) gene is well known for its crucial role in cell adhesion and the Wnt-signaling pathway. Previous studies have shown that gain-of-function mutations in the *CTNNB1* gene contribute to the occurrence and development of a variety of carcinomas in humans. Recently, de novo, heterozygous, loss-of-function mutations of the *CTNNB1* gene were found that partially explain intellectual disability in some patients. Other major clinical symptoms in these patients included microcephaly, abnormal facial features, motor delays, speech impairments, and deformities of the hands and feet. In addition, approximately 75% of these patients had mild visual defects, such as astigmatism, hyperopia, or strabismus

**Patient concerns:**

A 15-month-old Chinese boy, presenting with retinal detachment, lens and vitreous opacities, hypertonia of the extremities, mild thumb adduction, microcephaly, and developmental delay, is described. Targeted gene sequencing using an ophthalmic gene panel was performed to test for familial exudative vitreoretinopathy; however, the pathogenic gene was not found.

**Interventions:**

Genomic DNA analysis was performed to search for causing mutations.

**Diagnoses and outcomes:**

Whole-exome sequencing revealed a novel nonsense variation in exon 11 of the *CTNNB1* gene (c.1672C>T, p.Gln558X). Sanger sequencing of the patient and his parent confirmed this mutation and demonstrated it to be de novo. To the best of our knowledge, this is the first case report of a loss-of-function mutation of the *CTNNB1* gene in an Asian population.

**Lessons:**

Severe ophthalmic phenotype has not well been connected with loss of functional mutation of *CTNNB1* gene. Our finding expands the mutant spectrum of *CTNNB1* gene and adds new understanding of the phenotype.

## 1 Introduction

The *CTNNB1* gene, also known as β-catenin, encodes an adherens junction protein to mediate adhesion between cells by forming a link between cadherins and the actin cytoskeleton, which is critical for the establishment and maintenance of epithelial layers. In addition to its role in the cell–cell adhesion, β-catenin serves as a coactivator in the Wnt-signaling pathway, which plays essential roles in a multitude of developmental and homeostatic processes.^[1–3]^ The CTNNB1 protein has dual functions due to its unique structure, which contains 12 central armadillo repeats motif flanked by the C-terminal transactivation domain beginning with a Helix-C motif and an N-terminus that mediates degradation of the protein.^[4]^ It has been demonstrated that conditional loss- and gain-of-function mutations in β-catenin result in various phenotypes in distinct organs in mice, and somatic gain-of-function mutations in β-catenin are highly associated with tumorigenesis and tumor metastasis in humans.^[5,6]^ More recently, loss of function mutations in *CTNNB1* were found to be associated with human disease in a cohort of 765 patients with intellectual disabilities (IDs).^[7]^ Subsequently, several single case reports, as well as a study of 16 patients with *CTNNB1* de novo mutations, further confirmed that the *CTNNB1* gene is a pathogenic gene associated with ID and contributes to several other phenotypes, including microcephaly, abnormal facial features, hand and foot deformities, motor delays, speech delays, truncal hypotonia, peripheral hypertonia, behavioral abnormalities, and visual defects.^[8–11]^ The present study describes a de novo mutation of the *CTNNB1* gene in a 15-month-old Chinese boy who presented with retinal detachment, lens and vitreous opacities, microcephaly, and developmental delay.

## 2 Consent

The study protocol was approved by the ethics committee of Shanghai Children's Medical Center, and informed consent was obtained from the family of each patient.

## 3 Case report

The patient is a 15-month-old male, born from nonconsanguineous and healthy Chinese parents, without family history of birth defects, developmental delay, and/or IDs. He was delivered by cesarean section at 40-week gestation, had a birth weight of 3.6 kg, and was the first child of the couple. Neonatal clinical examination at the local hospital did not reveal any abnormalities.

At 3 months of age, he underwent an ophthalmologic examination after his parents noted that his eyes did not react to light. Results of an ultrasound of the eyes showed a retinal detachment and lens and vitreous opacities in both eyes. Intraocular pressures were as follows: oculus dexter: 15.3 mm Hg; oculus sinister: 14.7 mm Hg. Falciform retinal folds and fundus hemorrhages were also reported. Because familial exudative vitreoretinopathy was initially suspected, targeted gene sequencing using an ophthalmic gene panel was performed; however, the pathogenic gene was not found. Fundus fluorescein angiography in both eyes was normal. Ultrasound examination of the eyes at 4 and 7 months revealed persistent retinal detachments in both eyes.

At 9 months of age, the patient was 73.0-cm tall, weighed 8.0 kg, had a head circumference of 42.0 cm (<−2 standard deviation [SD]), but could not roll or sit by himself. He was also noted to have mild thumb adduction and hypertonia of the extremities.

At the age of 15 months, the patient was taken to the Medical Genetics Department at Shanghai Children's Medical Center, Shanghai Jiaotong University School of Medicine (Shanghai, China) to obtain a definitive diagnosis. Physical examination showed that he was 80.3-cm tall, weighed 10.0 kg, and head circumference of 44.0 cm (<−2SD). He had hypertonia, low-set ears, and high palate arch. He still had no reaction to light and appeared to have a complete lack of vision. He could raise his head but still could not sit or walk without support. No language development was found. Magnetic resonance imaging of the patient's brain, electroencephalogram, abdominal ultrasound, and hearing examination revealed no abnormalities. His genetic/metabolic workup, including karyotype, liver function tests, alkaline phosphatase, glucose, lactate, electrolytes, and urinalysis were all normal.

**Figure 1:**
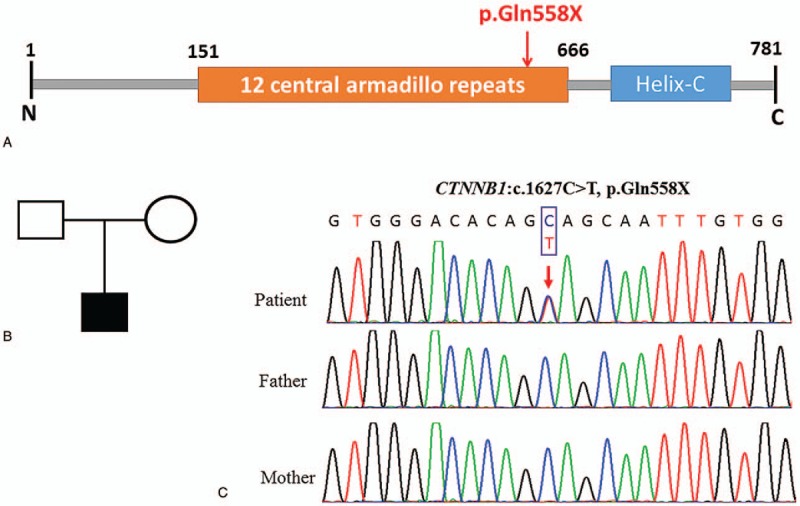
A de novo mutation was identified in the *CTNNB1* gene. (A) The schematic diagram of the mutant amino acids site, cylinders represent the 12 central armadillo repeats motif; (B) the family tree; (C) sequences showed a heterozygous nonsense mutation (c.1627C>T, p.Gln558X in exon 11) in the patient, and the parents were normal. Red arrows, mutant bases.

At this point of the investigation, we concluded a rare inherited syndrome of the patient. The patient was screened for causal variants using whole-exome sequencing (WES) as previously described by our own lab.^[12]^ Base calling and sequence read quality assessment were performed using Illumina HCS 2.2.58 software (Illumina, Inc., San Diego, CA) with the new versions of HiSeq control software and Real Time Analysis. The reads were mapped to the reference human genome (Human 37.3; SNP135) using NextGENe software (SoftGenetics LLC, State College, PA). WES generated a total of 34,350 variants. The Ingenuity Variant Analysis (Ingenuity Systems, Mountain View, CA) was used to analyze the candidate variants. The following variants were initially excluded: the common variants with the minor allele frequency greater than 1% in the control databases, including 1000 Genomes Project, the National Heart, Lung and Blood Institute Exome Sequencing Project, and the Exome Aggregation Consortium; the benign variants, including synonymous, harmless missense predicted by PolyPhen-2 (Cambridge, MA) and SIFT software, and those predicted to have no impact on splicing by MaxEntScan (Cambridge, MA). Subsequently, clinical symptoms of developmental delay, ophthalmic diseases, and microcephaly subsequently served as filtering indexes to analyze the candidate variants. Finally, a novel heterozygous nonsense variation (c.1627C>T, p.Gln558X) in exon 11 of the *CTNNB1* gene was identified in the affected individual. Sanger sequencing was used to examine the proband and parents in order to further confirm the WES results and to assess whether the identified variation was present in either of the parents. The Sanger sequencing results confirmed the presence of the *CTNNB1* gene variant in the patient and showed that the parents were normal **(**Fig. 1**)**, indicating that the variant was de novo.

## 4 Discussion

The present case involves a 15-month-old Chinese boy with a complex phenotype, which included retinal detachment, lens and vitreous opacities, hypertonia of the extremities, mild thumb adduction, microcephaly, and developmental delay. WES of the patient revealed a novel heterozygous nonsense mutation in exon 11 of the *CTNNB1* gene (c.1627C>T, p.Gln558X), which was demonstrated to be de novo via Sanger sequencing. The mutant of c.1627C>T was classified as pathogenic (PVS1+PS2+PM2), based on the American College of Medical Genetics and Genomics/the Association for Molecular Pathology variant-interpretation guidelines.^[13]^

**Figure 2:**
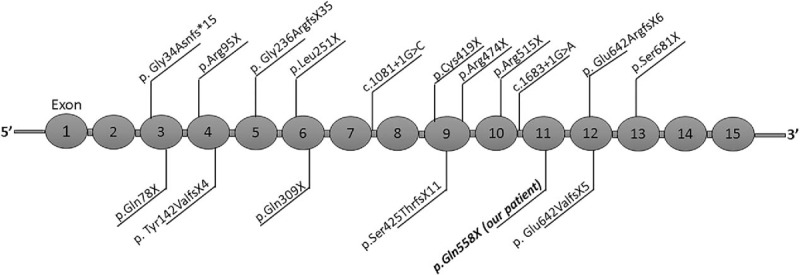
The schematic diagram of the distribution of 16 reported mutations as well as p.Gln558X in *CTNNB1* gene.

The *CTNNB1* gene is located at 3p22.1 (chr3:41240942–41281939) and contains 15 exons, which encodes a protein consisting of 781 amino acids (data from UCSC database, http://genome.ucsc.edu). CTNNB1 belongs to the armadillo family of proteins that are involved in genetic programs that control embryonic development and adult homeostasis and play an important role in the Wnt-signaling pathway and cell adhesion. The p.Thr551Met mutation, identified in an autism spectrum disorders (ASD) cohort study, was the first de novo mutation in the *CTNNB1* gene that was found to be connected to ID.^[14]^ However, the pathogenicity of this mutation was not well recognized due to a lack of functional studies and few cases with supporting evidence. Subsequently, 3 de novo loss of function mutants were found in ID patients demonstrating that germline mutations of the *CTNNB1* gene could lead to Mendelian inherited disease, which had a phenotype included in the Online Mendelian Inheritance in Man (OMIM) database (OMIM# 615075). To date, 20 loss-of-function mutations of the *CTNNB1* gene have been reported in 21 patients,^[7–11]^ including 9 nonsense, 7 frameshift, 2 splice, and 2 whole-gene deletions. The mutants are distributed in exons 3, 4, 5, 6, 9, 10, 12, 13, and introns 7 and 10 (Fig. 2**)**. Two missense mutations (p.Thr551Met in exon 11 and p.Leu388Pro in exon 8) were not listed in the table due to a lack of evidence for loss of function.

Most of the phenotypic characteristics of the patient in the present study were consistent with other reported cases in the literature **(**Table 1**)**. Although the child was too young to perform the related tests, his parents realized that his intelligence was below the level of his peers. Another patient, with a mutation in exon 11 of *CTNNB1* (p.Thr551Met), presented with ASD, moderate ID, though no further clinical data were provided.^[14]^ Consequently, a meaningful comparison of clinical feature could not be conducted. The most significantly different feature is that approximately 75% of patients with *CTNNB1* loss of function mutations have mild visual defects, mostly characterized by astigmatism, hyperopia, or strabismus,^[10]^ while the patient in this study suffered from severe ophthalmic disease resulting in complete loss of vision. Wnt/β-catenin signaling is essential in vertebrate eye development and conditional loss-of-function mutations of β-catenin in mice can lead to abnormal lens morphology and retinal anomalies^[6,15]^; however, the severe eye disease observed in the patient in this study was rarely described in humans with *CTNNB1* loss of function mutations. Moreover, approximately 5% of patients with a molecular diagnosis have multiple molecular diagnoses, which involve more than 1 clinical diagnosis, and cause more serious phenotypic symptoms.^[16]^ Taking this into account, the WES data were reanalyzed with only the ophthalmic disease phenotype in order to determine whether other pathogenic genes were responsible for or aggravated the eye symptoms in the patient. In total, 11 heterozygous variations were found. Of these, 10 were recessively inherited genes (*USH2A*, *SLC19A3*, *RARS2*, *HPS1*, *PRSS1*, *PEX5*, *UBR1*, *NDE1*, *TYK*, and *TRIOBP*), and 1 was a missense mutation in the *KMT2D* gene (p.Arg3707Gln), which was present in his mother, though she lacked similar clinical features, indicating that the mutation was benign. Therefore, it appears that the *CTNNB1* gene mutant was responsible for the clinical features of the patient in the present study. *CTNNB1* should be added to the growing ophthalmic disease list when designing an ophthalmic gene panel.

**Table 1:**
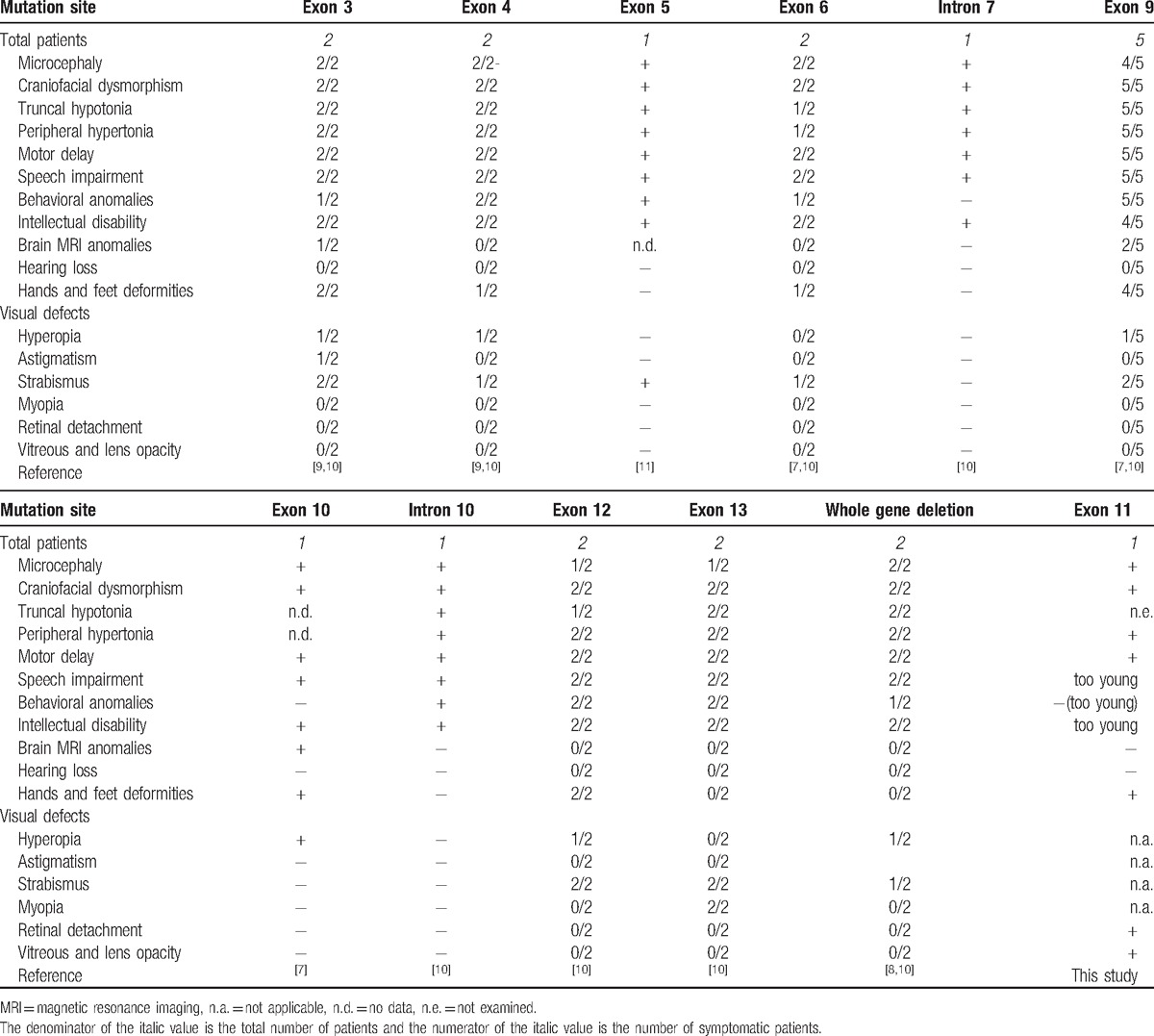
Clinical manifestations of patients carrying loss of function mutations in *CTNNB1* gene.

In conclusion, the present study reported on a Chinese patient with a novel de novo heterozygous nonsense mutation in *CTNNB1* gene (p.Gln558X). The patient had a severe ocular phenotype, which had not been well recognized. Different analyses of the WES data suggested that no other pathogenic gene contributed to the phenotype. This study expands the mutant spectrum of the *CTNNB1* gene and enhances the understanding of this phenotype. To the best of our knowledge, this is the first case report of a loss-of-function mutation of the *CTNNB1* gene in Asia.
